# E2 ubiquitin-conjugating enzyme UBE2L6 promotes *Senecavirus A* proliferation by stabilizing the viral RNA polymerase

**DOI:** 10.1371/journal.ppat.1008970

**Published:** 2020-10-26

**Authors:** Liang Li, Juan Bai, Hui Fan, Junfang Yan, Shihai Li, Ping Jiang

**Affiliations:** 1 Key Laboratory of Animal Diseases Diagnostic and Immunology, Ministry of Agriculture, MOE International Joint Collaborative Research Laboratory for Animal Health & Food Safety, College of Veterinary Medicine, Nanjing Agricultural University, Nanjing, China; 2 Jiangsu Co-innovation Center for Prevention and Control of Important Animal Infectious Diseases and Zoonoses, Yangzhou, China; University of Maryland, UNITED STATES

## Abstract

*Senecavirus A* (SVA), discovered in 2002, is an emerging pathogen of swine that has since been reported in numerous pork producing countries. To date, the mechanism of SVA replication remains poorly understood. In this study, utilizing iTRAQ analysis we found that UBE2L6, an E2 ubiquitin-conjugating enzyme, is up-regulated in SVA-infected BHK-21 cells, and that its overexpression promotes SVA replication. We determined that UBE2L6 interacts with, and ubiquitinates the RNA-dependent RNA polymerase of SVA, (the 3D protein) and this ubiquitination serves to inhibit the degradation of 3D. UBE2L6-mediated ubiquitination of 3D requires a cystine at residue 86 in UBE2L6, and lysines at residues 169 and 321 in 3D. Virus with mutations in 3D (rK169R and rK321R) exhibited significantly decreased replication compared to wild type SVA and the repaired viruses, rK169R(R) and rK321R(R). These data indicate that UBE2L6, the enzyme, targets the 3D polymerase, the substrate, during SVA infection to facilitate replication.

## Author summary

*Senecavirus A* (SVA) is a newly emerging pathogen causing swine idiopathic vesicular disease and epidemic transient neonatal losses. Infections have been reported in many pork producing countries, yet the mechanism of SVA replication remains poorly understood. In this study, we found that UBE2L6, an E2 ubiquitin-conjugating enzyme, is up-regulated in SVA-infected BHK-21 cells. The viral RNA dependent RNA polymerase (RdRp) 3D is ubiquitinated by UBE2L6, and the lysines at residues 169 and 321 of 3D are the required ubiquitination sites. The level of replication of recombinant viruses harboring ubiquitination-deficient 3D was significantly decreased compared to parental SVA. Our data demonstrate that UBE2L6 ubiquitinates SVA 3D, thereby facilitating SVA infection. These results may make it possible to identify novel targets for disease treatment.

## Introduction

*Senecavirus A* (SVA), previously designated Seneca Valley virus (SVV), is a non-enveloped single-stranded RNA virus in the genus *Senecavirus* within the Picornaviridae family. SVA is an emerging pathogen of swine [1]; and since 2014 SVA associated vesicular disease has been reported in Canada [2], the United States [3], Colombia [4], Brazil [5–7], and China [8–11]. The genome of SVA is about 7.2 kb in length and contains one large open reading frame (ORF) that encodes a polyprotein. During viral replication, the polyprotein is cleaved into 4 structural proteins, 1A (VP4), 1B (VP2), 1C (VP3), 1D (VP1), and 7 non-structural proteins, 2A, 2B, 2C, 3A, 3B (Vpg), 3C and 3D (RNA-dependent RNA polymerase, RdRp). The non-structural proteins are responsible for genome replication and assembly.

The ubiquitin-proteasome system (UPS) is involved in post-translational modification of proteins, and regulates a variety of cellular processes, including cell proliferation, apoptosis, and immune signaling. It also plays a role in viral infection, often by being utilized for viral replication [12–17]. For example, ubiquitination is required for coxsackievirus B3 replication in HeLa cells [18]. Nsp2 and Nsp11 of porcine reproductive and respiratory syndrome virus (PRRSV) utilize the host’s UPS to interfere with the polyubiquitination of IκBα, thereby inhibiting the activation of NF-κB and the production of type I interferon [19,20].

UBE2L6 (also known as UbcH8 [21]) is an E2 ubiquitin/ISG15-conjugating enzyme that plays a determinative role in targeting c-Myc for proteasomal degradation by interacting with E3 ubiquitin ligase, thereby suppressing cell proliferation and xenograft tumor growth [22]. UBE2L6 also plays an important role in transducing DNA damage signals by interacting with E3 ubiquitin ligase RNF8 [23–25]. UBE2L3, the E2 enzyme most closely related to UBE2L6, induces the degradation host antiviral protein A3A, thereby maintaining HBV cDNA [26]. The mechanism by which UBE2L6-mediated ubiquitination functions in viral infection is not well understood.

To explore the mechanism of SVA infection, we performed an iTRAQ (isobaric tags for relative and absolute quantification) analysis on uninfected and SVA-infected ST cells. We found that UBE2L6 is significantly upregulated in infected cells, and it interacts with the SVA 3D protein and stabilizes it through polyubiquitination. We also found that cysteine residue 86 of UBE2L6 is required for ubiquitination of 3D, and that lysine residues 169 and 321 of 3D are the corresponding ubiquitination sites.

## Results

### iTRAQ analysis of protein expression in SVA-infected ST cells

**Fig 1:**
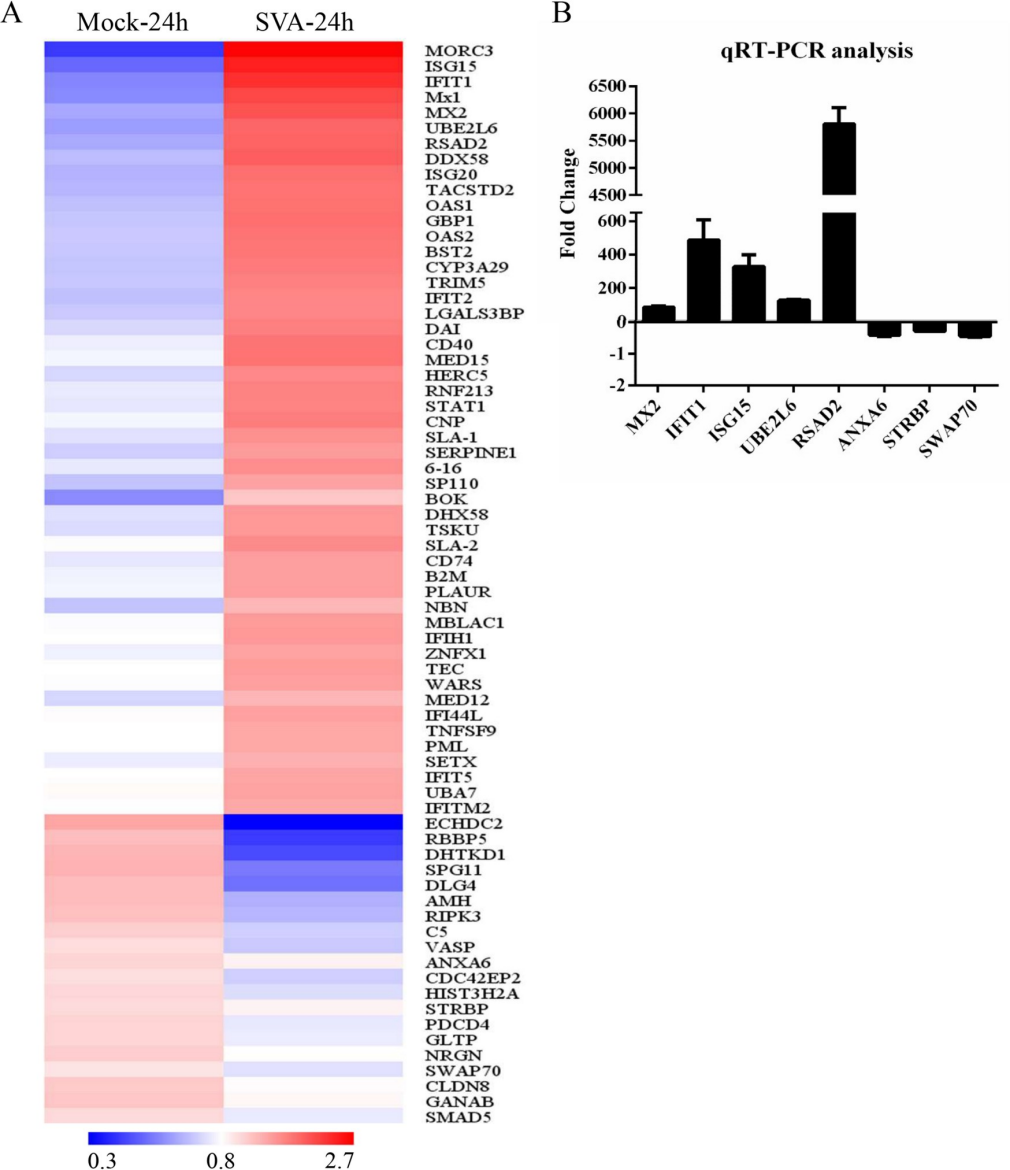
iTRAQ analysis of SVA-infected ST cells. ST cells were mock- or SVA-infected at MOI 0.1 and harvested at 12 and 24 hpi for proteomic analysis. (**A**) Cluster analysis of the 50 upregulated and 20 downregulated genes with the greatest fold change values. Red indicates increased (infected/mock) gene expression, and blue indicates decreased expression. (**B**) Validation of iTRAQ data by qRT-PCR analyses. *Mx2*, *IFIT1*, *ISG15*, *UBE2L6*, and *RSAD2* mRNA levels were upregulated, and *ANXA6*, *STRBP*, and *SWAP70* mRNA levels were downregulated. Values are presented as means ± SD from three independent experiments.

To investigate the host proteins and pathways involved in SVA replication, iTRAQ analysis was performed on SVA infected and uninfected ST cells. After analysis of samples obtained at 24 hpi, we selected the 50 proteins exhibiting the largest increases in abundance (infected/uninfected), and the 20 proteins with the greatest shift in the opposite direction (Fig 1A). The 50 proteins in the first category were considered to be “up-regulated” while the other 20 were “down-regulated”. qRT-PCR analysis of the 5 up-regulated genes corresponding to proteins with the greatest fold change (Mx2, IFIT1, ISG15, UBE2L6, and RSAD2), and the 3 genes corresponding to down-regulated proteins with the greatest fold change (ANXA6, STRBP, and SWAP70) was consistent with the proteomics analysis, demonstrating that the iTRAQ results are reliable (Fig 1B).

### Screening of the host proteins

**Fig 2:**
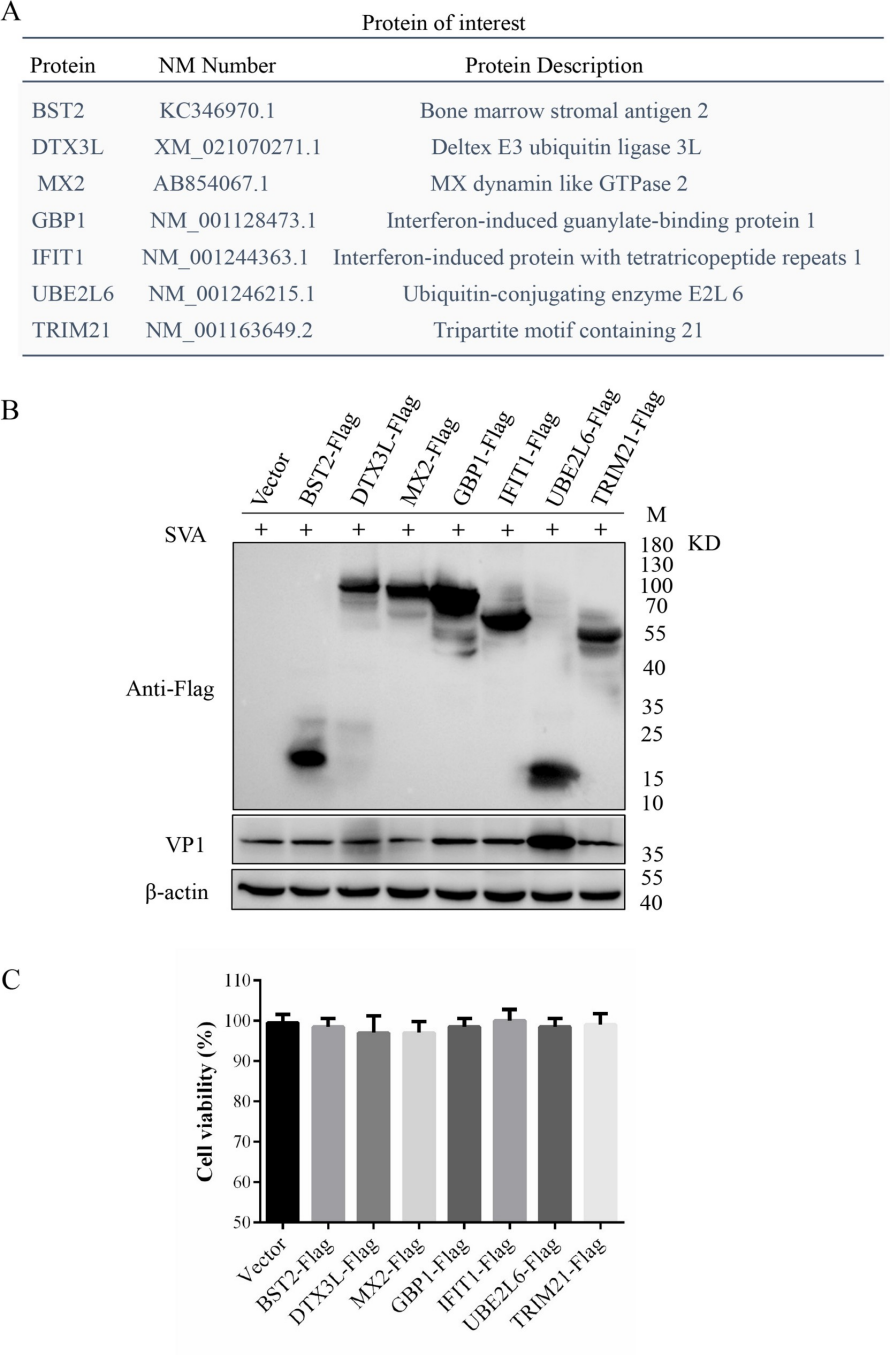
Effects of differentially expressed proteins on SVA replication. (**A**) Host proteins involved in SVA replication. (**B**) Western blot of transfected BHK-21 cells challenged with SVA for 16 h. (**C**) Viability of cells overexpressing the indicated genes. Values are presented as the mean ± SD from three independent experiments.

Proteins BST2, DTX3L, MX2, GBP1, IFIT1, UBE2L6 and TRIM21 were selected for further analysis (Fig 2A) and the corresponding genes were cloned into pCAGGS-Flag vectors. The constructs were transfected into BHK-21 cells, which were then infected with SVV-CH-SD at 0.01 MOI for 16 hours. Western blotting for VP1 showed that UBE2L6-transfected cells had significantly increased levels of SVA replication, and MX2 transfected cells had weakly-suppressed SVA replication (Fig 2B). Cell viability assays demonstrated the uniformity of cell growth and activity of all transfected cells (Fig 2C).

### UBE2L6 overexpression promotes SVA replication

**Fig 3:**
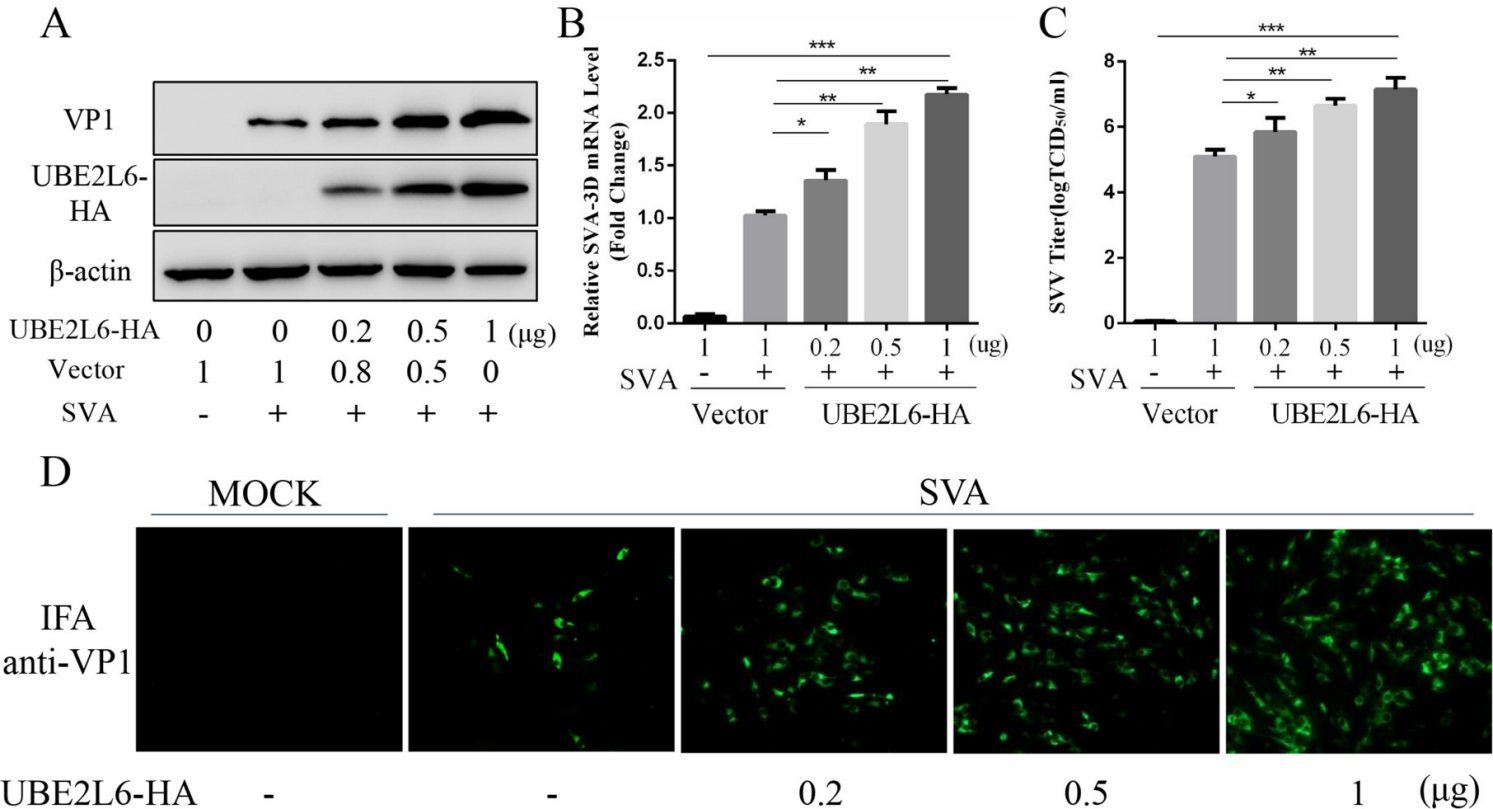
UBE2L6 overexpression promotes SVA replication. (**A**) Western blot of pUBE2L6-HA and/or empty vector (Vec) transfected BHK-21 cells challenged with SVA for 16 h. VP1 and UBE2L6 were probed with anti-VP1 and anti-HA antibodies. (**B**) Viral RNA levels were quantitated using qRT-PCR. (**C**) Virus yields in infected cell supernatants presented as TCID_50_ per milliliter. (**D**) IFA of SVA infected cells. All samples run in triplicate. *, P < 0.05; **, P < 0.01.

BHK-21 cells were transfected with pUBE2L6-HA and/or empty vector for 24 h and then infected with SVA at MOI of 0.01. Cell extracts were assayed by Western blot and qRT-PCR to measure levels of SVA protein and transcription. The results showed that UBE2L6 overexpression resulted in increased levels of viral VP1 and 3D mRNA in a UBE2L6-concentration-dependent manner (Fig 3A and 3B). TCID_50_ assays showed that titers of extracellular progeny virus increased significantly in response to UBE2L6 (Fig 3C), and indirect immunofluorescence assays demonstrated that levels of intracellular SVA, as determined by VP1-specific fluorescence, increased with UBE2L6 concentration (Fig 3D).

### Knockout of UBE2L6 suppresses SVA replication

**Fig 4:**
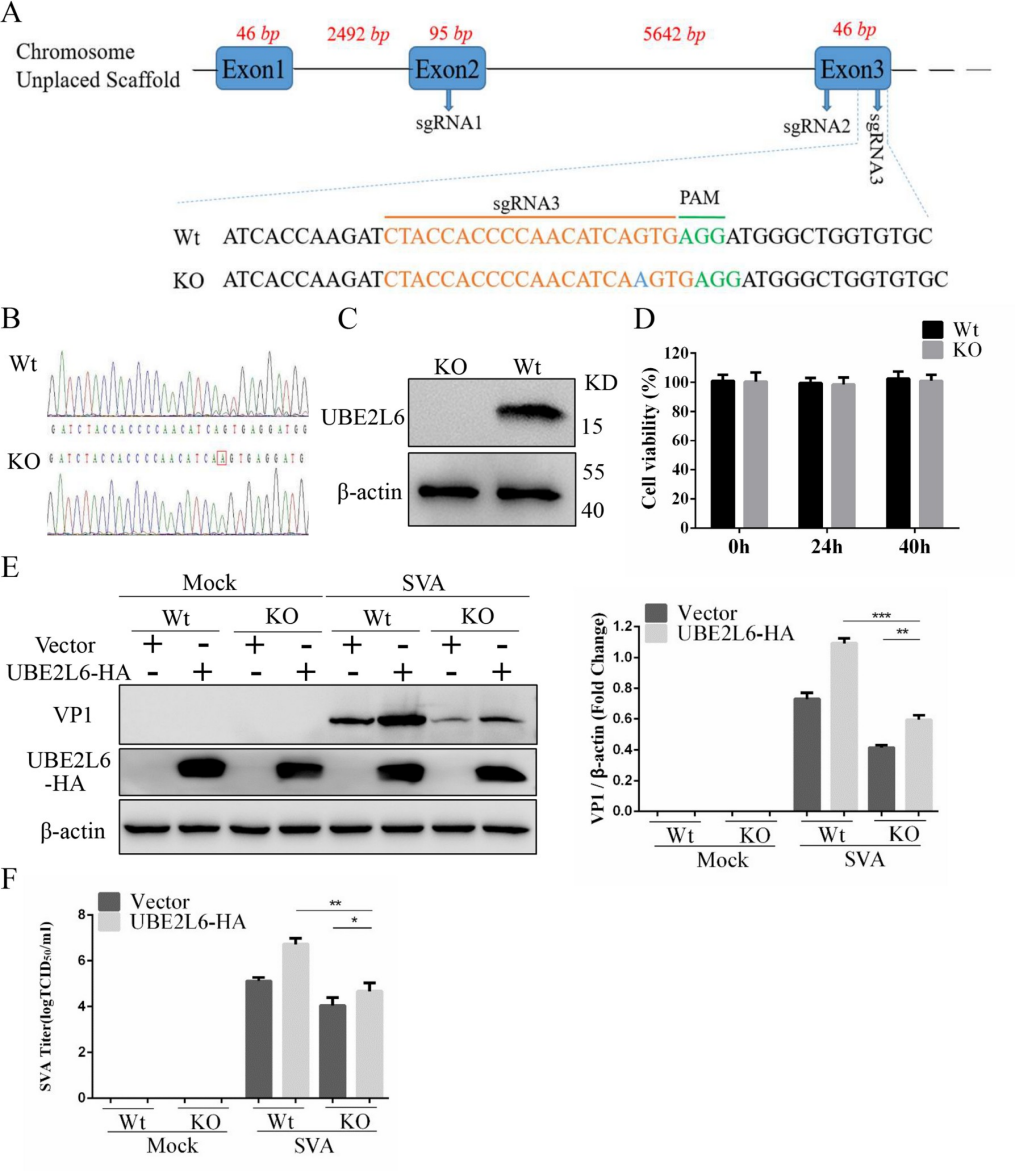
Knockout of UBE2L6 suppresses SVA replication. (**A**) Sequences in *UBE2L6* targeted by sgRNAs 1–3. AGG (green) is the protospacer adjacent motif (PAM). The highlighted base (blue) indicates an indel. (**B**) Sanger sequencing showing the indel in *UBE2L6*. (**C**) Western blot analysis of UBE2L6 expression in the BHK-UBE2L6-KO and BHK-Wt cells. β-actin was used as a loading control. (**D**) Viability assay of BHK-UBE2L6-KO and BHK-Wt cells. 2× 10^4^ cells were seeded into wells of 96 well plates and viability were measured at 0 h, 24 h and 40 h. (**E-F**) BHK-UBE2L6-KO and Wt cells were transfected with pCAGGS-UBE2L6-HA or empty vector then infected with SVA (MOI 0.01) for 16 h. Levels of VP1 (**E**) and virus titers (**F**) were determined by Western blot and TCID_50_ respectively. Data are expressed as the means ± SD from three independent experiments. *, P < 0.05; **, P < 0.01.

The role of UBE2L6 in SVA replication was further investigated using UBE2L6 knock-out BHK-21 cells (BHK-UBE2L6-KO) (Fig 4A). Sanger sequencing confirmed that the UBE2L6 sequence had been disrupted successfully (Fig 4B) and Western blotting showed that UBE2L6 levels in the knock out cells were below the limits of detection (Fig 4C). BHK-UBE2L6-KO and BHK-Wt cell viability was indistinguishable (Fig 4D). Subsequently, the replication efficiency of SVA was assessed in BHK-UBE2L6-KO and BHK-Wt cells. SVA replication levels in KO cells was significantly reduced compared to Wt as determined by Western blotting for VP1 and TCID_50_ assay. Overexpression of UBE2L6 in the knockout cells partially restored SVA replication (Fig 4E). These results suggest that UBE2L6 is essential for SVA replication.

### Interaction of SVA proteins with UBE2L6

**Fig 5:**
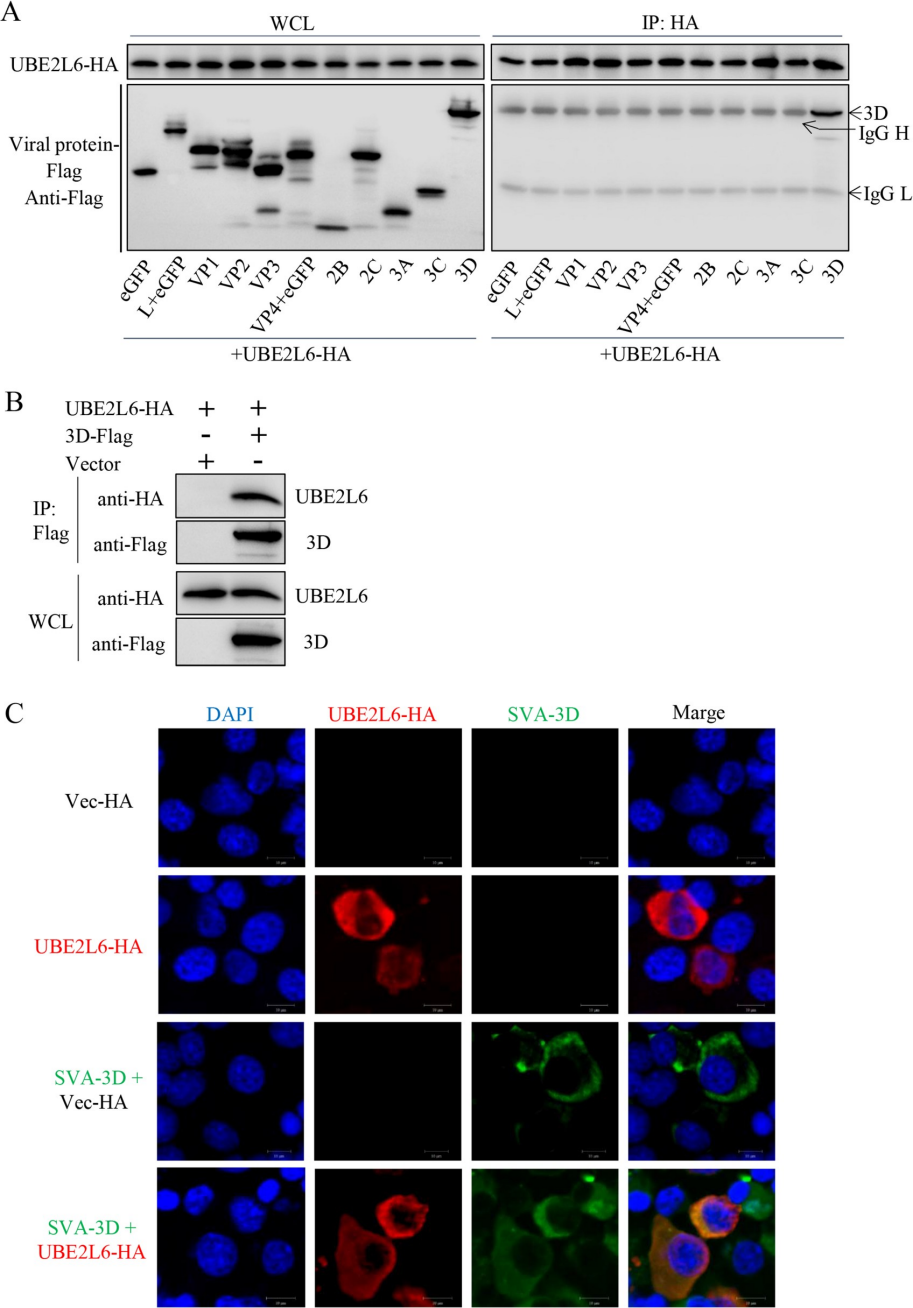
UBE2L6 interacts with SVA 3D. (**A**) Immunoprecipitation and Western blot of BHK-21 cells cotransfected with Flag-tagged SVA proteins and HA-tagged UBE2L6; proteins immunoprecipitated with rabbit anti-HA. (**B**) Western blot showing that the UBE2L6-3D interaction is specific. p3D-Flag and pUBE2L6-HA cotransfected cell lysates were collected, and then co-IP was performed using mouse anti-Flag. (**C**) Colocalization of UBE2L6 and viral 3D. Confocal microscopy of BHK-21 cells transfected with pUBE2L6-HA then infected with SVA (MOI 0.01) for 16 h. SVA 3D (green), pUBE2L6-HA (red), and nuclei (blue). Experiments were performed in triplicate.

Eleven recombinant plasmids were constructed to express SVA proteins in BHK-21 cells. Nine were tagged with the FLAG epitope (VP1, VP2, VP3, 2B, 2C, 3A, 3C, and 3D) and two with EGFP (L and VP4) because their genes were too short to support a FLAG-tag. The constructs were transfected into BHK-21 cells together with pUBE2L6-HA. pCAGGS (expressing EGFP and FLAG) was used as a negative control. Co-immunoprecipitation assays showed that UBE2L6 interacts only with SVA 3D (Fig 5A and 5B), and confocal microscopy showed that UBE2L6 and 3D colocalize in the cytoplasm of the SVA-infected cells (Fig 5C). Together, these data demonstrate that UBE2L6 interacts with the 3D protein of SVA.

### The viral 3D protein is ubiquitinated during SVA infection

**Fig 6:**
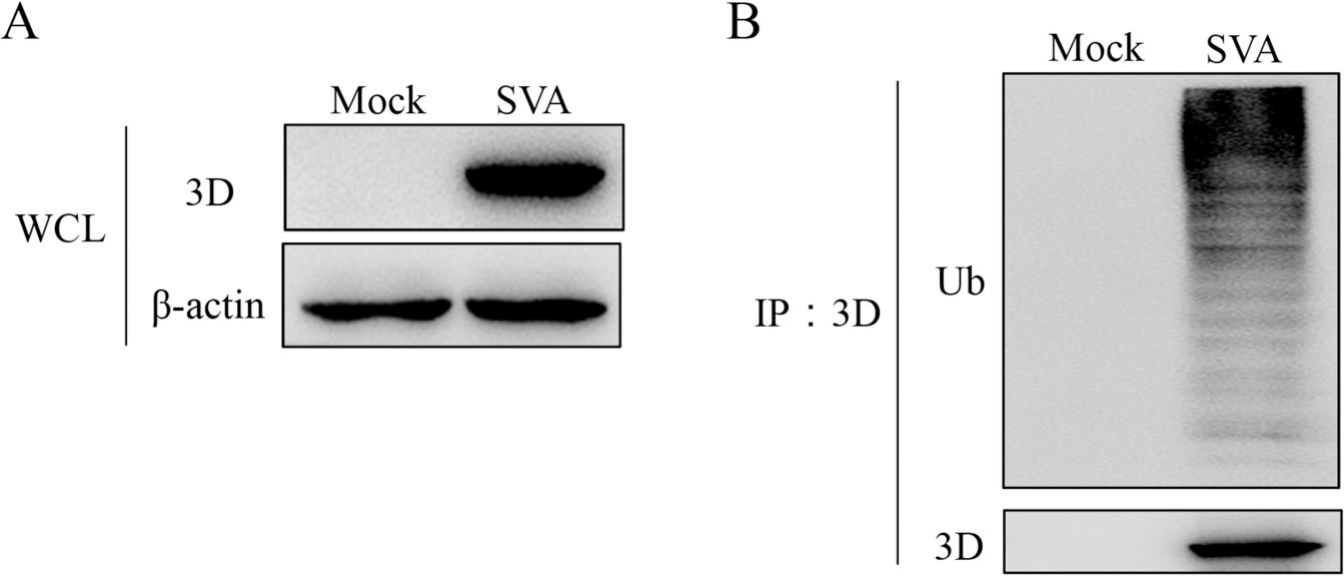
SVA 3D is ubiquitinated during infection. BHK-21 cells (1 × 10^7^) were infected with SVA (MOI 1) and harvested at 7 h post infection before immunoprecipitation with an anti-3D antibody. IP and IB analyses were performed with the indicated antibodies. (**A**) Western blot of infected cell lysates. (**B**) Immunoprecipitation with anti-3D antibody, and Western blotting with anti-Ub, and anti-3D antibodies, of infected cells lysates.

Because UBE2L6 is an E2 ubiquitin-binding enzyme, we conducted an experiment to determine if UBE2L6 exerts its effects during SVA infection via the ubiquitination modification pathway. SVA infected BHK-21 cells (MOI 1) were assayed by Western blot at IP 7 hpi. Western blotting was initially used to determine the level of 3D in cell lysates (Fig 6A). Immunoprecipitation with SVA 3D monoclonal antibody and subsequent blotting with Ub endogenous antibody revealed specific precipitation of the 3D protein and ubiquitination of 3D (Fig 6B). These results indicate that SVA 3D is ubiquitinated during infection.

### UBE2L6 mediates polyubiquitination of the viral 3D protein

**Fig 7:**
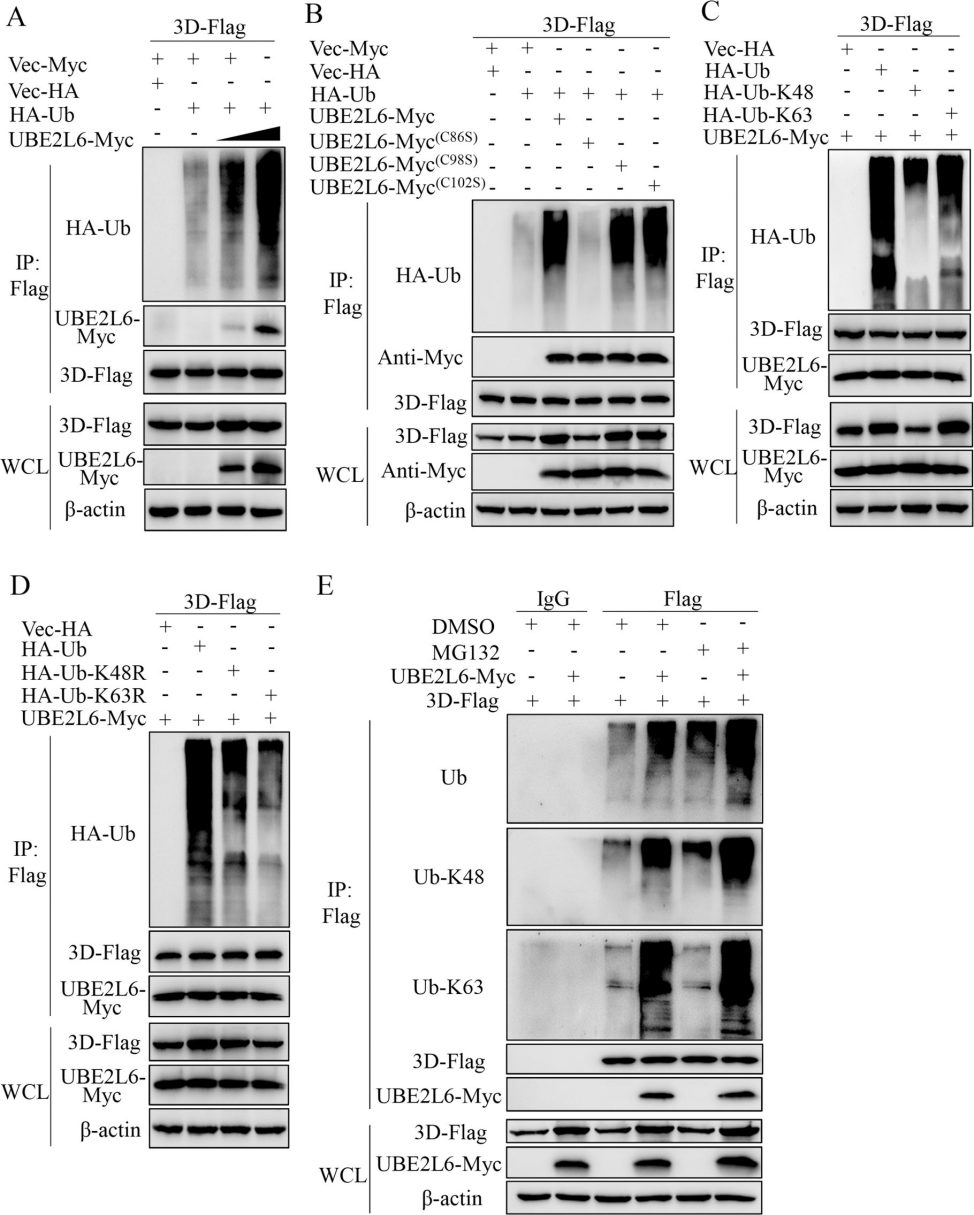
UBE2L6 mediates ubiquitination of 3D. (**A**) pUBE2L6 ubiquitinates 3D in a concentration-dependent manner. HEK-293T cells, cotransfected with p3D-Flag and pUBE2L6-Myc (0, 1, or 2 μg), were subjected to co-IP with anti-Flag (MAb), then Western blotting with anti-HA, anti-Flag and anti-Myc MAbs. (**B**) The cysteine at position 86 in UBE2L6, is necessary for 3D ubiquitination by UBE2L6. Cell lysates from HEK-293T cells cotransfected with p3D-Flag and pUBE2L6-Myc, pUBE2L6 (C86S), pUBE2L6 (C98S), or pUBE2L6 (C102S), together with pHA-Ub were subjected to IP and immunoblotting. (**C, D**) The residues K48 and K63 in 3D are necessary for UBE2L6-mediated polyubiquitination of 3D. HEK293T cells cotransfected with p3D-Flag, pUBE2L6-Myc, pHA-Ub, or pHA-Ub-K48 (only K48 of the seven Lys residues remained), pHA-Ub-K63 (only K63 of the seven Lys residues remained), pHA-Ub-K48R (pHA-Ub with mutation of K48 to R) or pHA-Ub-K63R (pHA-Ub with mutation of K63 to R), were subjected to IP and immunoblotting as described above. (**E**) HEK293T cells were cotransfected with p3D-Flag and pUBE2L6-Myc or vector for 24 h followed by treatment with 10 μM MG132 or DMSO for 6 h. Thirty hours later, the cell extracts were subjected to IP and immunoblotting by using antibodies to endogenous Ub, K48 and K63.

To determine whether UBE2L6 is involved in 3D ubiquitination, HEK-293T cells were cotransfected with p3D-Flag, pHA-Ub, and pUBE2L6-Myc (0, 1, or 2 μg). Immunoprecipitation and immunoblotting showed that 3D is ubiquitinated in a UBE2L6 concentration-dependent manner (Fig 7A). Next, we generated three pUBE2L6-Myc plasmids, each containing a single serine substitution (C86S, C98S, and C102S). Each plasmid was transfected into HEK-293T cells together with p3D-Flag and pHA-Ub and cell extracts were subjected to immunoprecipitation and immunoblotting. The data show that while 3D ubiquitination was somewhat decreased in cells transfected with the C98S and C102S mutants, 3D ubiquitination was markedly depressed in cells transfected with the C86S mutant (Fig 7B). To examine 3D polyubiquitination by UBE2L6 in detail, HEK-293T cells were co-transfected with p3D-Flag, pUBE2L6-Myc, and pHA-Ub or pHA-Ub mutant constructs. The mutants were pHA-Ub-K48 (of the seven Lys residues, only K48 remained), pHA-Ub-K63 (of the seven Lys residues, only K63 remained), pHA-Ub-K48R (pHA-Ub with a K48 to R substitution), or pHA-Ub-K63R (pHA-Ub with a K63 to R substitution). IP and immunoblotting showed that UBE2L6 mediated 3D polyubiquitination in cells transfected with HA-Ub-K48 and HA-Ub-K63 (Fig 7C). The levels of UBE2L6-mediated 3D polyubiquitination in cells transfected with HA-Ub-K48R and HA-Ub-K63R were significantly lower than in cells transfected with HA-Ub (Fig 7D). Additionally, endogenous K48/K63 branched ubiquitin chains were detected in cells transfected with UBE2L6 and 3D by using antibodies against Ub, Ub-K48, and Ub-K63. The results showed that transfection with a UBE2L6 expression construct increased levels of 3D ubiquitination by endogenous Ub, Ub-K48, and Ub-K63. MG132 treatment induced significantly higher levels of 3D ubiquitination by Ub and Ub-K48, but not by Ub-K63 (Fig 7E). Altogether, these results demonstrate that the K48- and K63-linked chains are involved in UBE2L6-mediated 3D polyubiquitination, and that this polyubiquitination depends on the cysteine at position 86.

### UBE2L6 stabilizes the viral 3D protein

**Fig 8:**
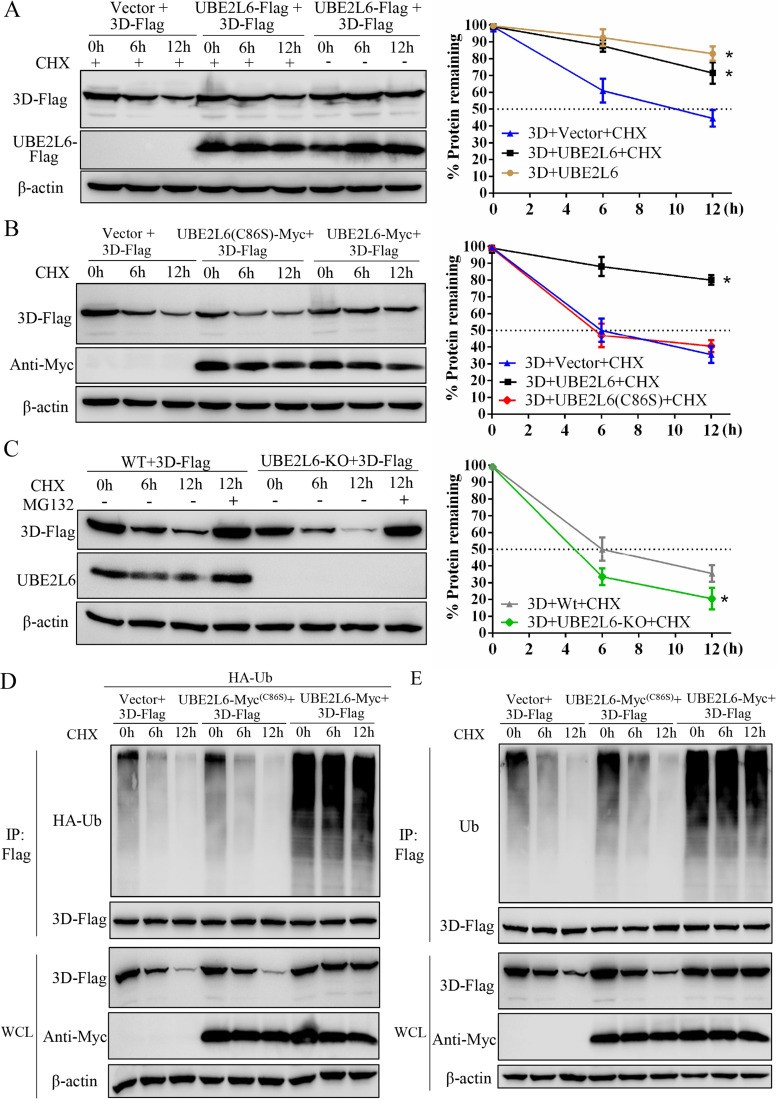
Ubiquitination affects the stability of 3D. (**A**) Western blot for 3D protein in BHK-21 cells cotransfected with pUBE2L6-Flag or empty vector, and p3D-Flag for 24 h then treated with cycloheximide (CHX). Band intensity was quantified using ImageJ, β-actin was used as loading control. The relative quantities of 3D are shown as percentages of 3D at 0 h. (**B**) Western blot for 3D protein in BHK-21 cells cotransfected with empty vector, pUBE2L6-Myc, or pUBE2L6(C86S), together with p3D-Flag for 24 h then treated with cycloheximide. (**C**) BHK-Wt cells or BHK-UBE2L6-KO cells were transfected with p3D-Flag for 24 h, designated 0 h, and then treated with CHX or MG132, as indicated, for up to 12 h. Cells were harvested and analyzed by Western blot with anti-Flag, anti-UBE2L6 and anti-β-Actin antibodies. (**D, E**) BHK21 cells cotransfected with empty vector, pUBE2L6-Myc, or pUBE2L6(C86S), p3D-Flag, together with pUb-HA (**D**) or without pUb-HA (**E**) for 24 h then treated with cycloheximide. And the levels of ubiquitination of 3D were evaluated by IP and immunoblotting. Data are expressed as the means ± SD from three independent experiments. *, P < 0.05; **, P < 0.01.

BHK-21 cells, transfected with p3D-Flag alone or together with pUBE2L6-Flag for 24 h were treated with CHX. Image analysis of Western blotting showed that levels of 3D protein in the p3D-Flag only group were significantly lower than 3D levels in the p3D-Flag plus pUBE2L6-Flag group 12 h after treatment. These results indicated that UBE2L6 acts to stabilize the viral 3D protein (Fig 8A). We repeated this experiment using the UBE2L6(C86S) mutant, and found that the levels of SVA 3D were significantly lower than in cells transfected with wild type UBE2L6 at 6 and 12 h after treatment (Fig 8B). BHK-UBE2L6-KO cells transfected with pCAGGS-3D-Flag for 24 h were treated with CHX together with or without MG132, a proteasome inhibitor. Western blotting showed that in the knockout cells, degradation of 3D was significantly greater than in wild type cells, and that MG132 stabilized 3D to the level observed in the cells before CHX treatment (Fig 8C). These data demonstrate that UBE2L6 acts to stabilize 3D levels (by approximately 20~40%) by preventing its proteasomal degradation.

To understand how ubiquitin contributes to UBE2L6-mediated 3D stability, BHK-21 cells were transfected with HA-Ub and 3D-Flag together with UBE2L6 or its mutant derivative UBE2L6(C86S) for 24 h and then treated with CHX. Image analysis of Western blotting showed that 12 hours after CHX treatment, levels of 3D ubiquitination in UBE2L6 transfected cells were higher than those in cells receiving the empty vector or in UBE2L6(C86S) transfected cells. The levels of 3D in empty vector and UBE2L6(C86S) transfected cells were 50~75% lower than levels in UBE2L6 transfected cells (Fig 8D). Additionally, IP and Western blotting with anti-Ub antibody showed that the changes in endogenous Ub levels were similar to those seen in the Ub-HA co-expression experiment (Fig 8E). These results demonstrate that ubiquitination by UBE2L6 stabilizes the viral 3D protein.

### K169 and K321 are the ubiquitination sites of SVA 3D

**Fig 9:**
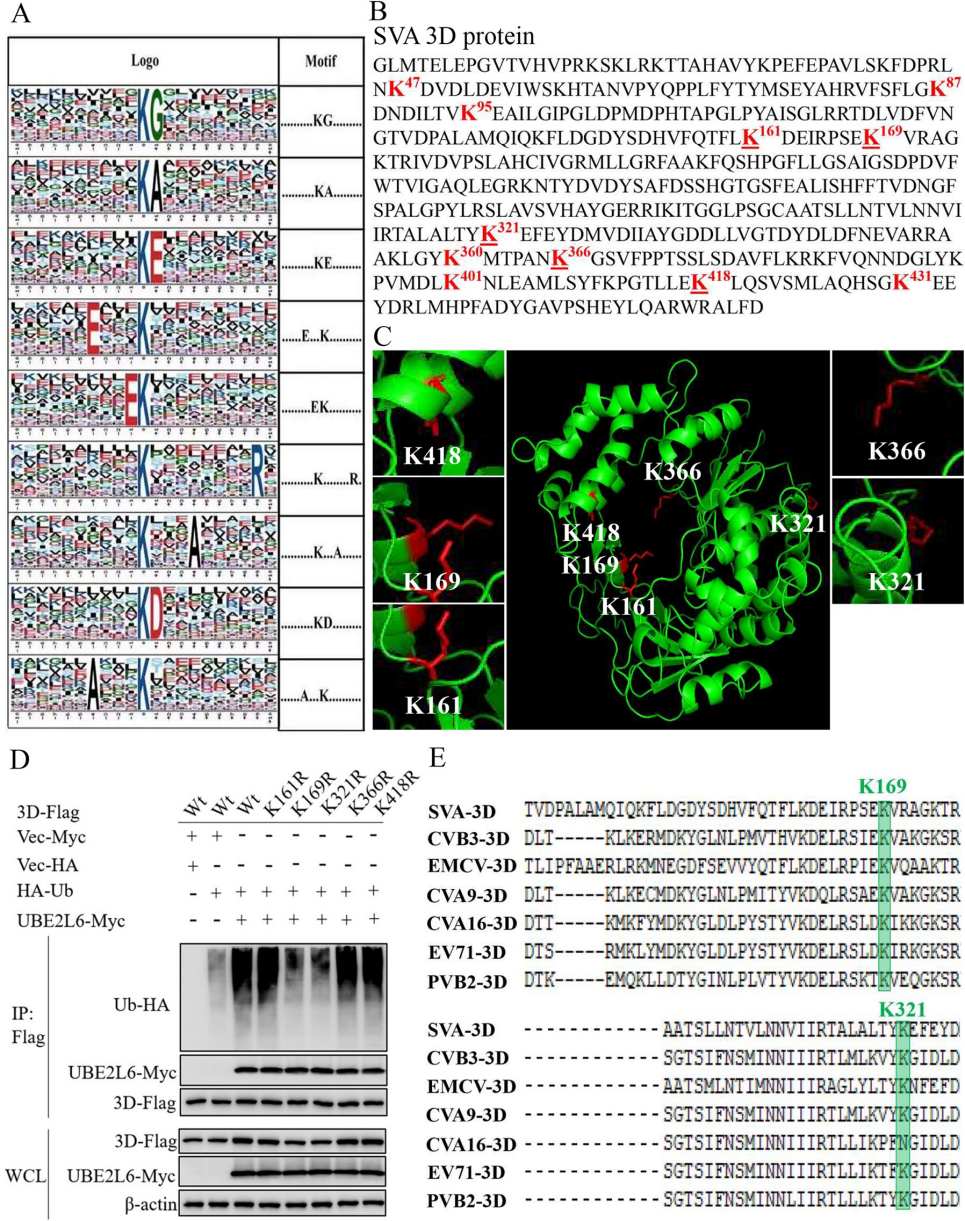
Lys169 and Lys321 of SVA 3D are major ubiquitination sites. (**A**) Ubiquitination motifs and the conservation of Ub sites. The height of each letter corresponds to the frequency of that amino acid residue at that position. The central K indicates ubiquitinated Lys. (**B**) Amino acid sequence of SVA 3D. The lysine (K) residues in the potential ubiquitination sites are shown in red, and the K residues corresponding to those in our mutants are underlined. (**C**) Predicted three-dimensional structure of 3D, SVA strain SVV-CH-SD, and the positions of mutated K residues. (**D**) Co-IP and Western blotting of HEK-293T cells cotransfected with pUBE2L6-Myc and p3D-FlagWt, K161R, K169R, K321R, K366R or K418R, together with pHA-Ub. Co-IP was done using anti-Flag MAb, and Western blotting was done with anti-HA, anti-Flag, and anti-Myc MAbs. (**E**) Alignment of 3D sequences from different viruses of Picornaviridae: Senecavirus A (SVA) (GenBank accession no. MH779611), coxsackievirus B3 (CVB3) (GenBank accession no. M16572), Encephalomyocarditis virus (EMCV) (GenBank accession no. HM641897), coxsackievirus A9 (CVA9) (GenBank accession no. D00627), coxsackievirus A16 (CVA16) (GenBank accession no. AY790926), enterovirus 71 (EV71) (GenBank accession no. AB204852) and poliovirus (PVB2) (GenBank accession no. KT353719). The selected K169 and K321 lysine residues are in green.

Ubiquitination occurs mainly at lysine residues. Bioinformatics analysis revealed ubiquitination motifs in the 3D protein (Fig 9A and 9B). I-TASSER (https://zhanglab.ccmb.med.umich.edu/I-TASSER/) was also used to predict protein structure (Fig 9C). Ubiquitination of 3D is predicted within or adjacent to the central region of the protein. To test this hypothesis we constructed five mutants, each with a single amino acid substitution in this region: pCAGGS-3D-Flag(K161R), K169R, K321R, K366R, and K418R. HEK-293T cells were cotransfected with pCAGGS-3D-Flag or the mutants, along with pHA-Ub and pUBE2L6-Myc. Co-IP results showed that only 3D-Flag(K169R) and K321R were not ubiquitinated by UBE2L6 (Fig 9D). Alignment analysis of polymerase sequences from different picornaviruses shows that the K169 and K321 sites of 3D are relatively conserved (Fig 9E). These results suggest that the K169 and K321 sites are necessary for UBE2L6 ubiquitination of SVA 3D.

### Construction of SVA 3D mutants and evaluation of their ubiquitination

**Fig 10:**
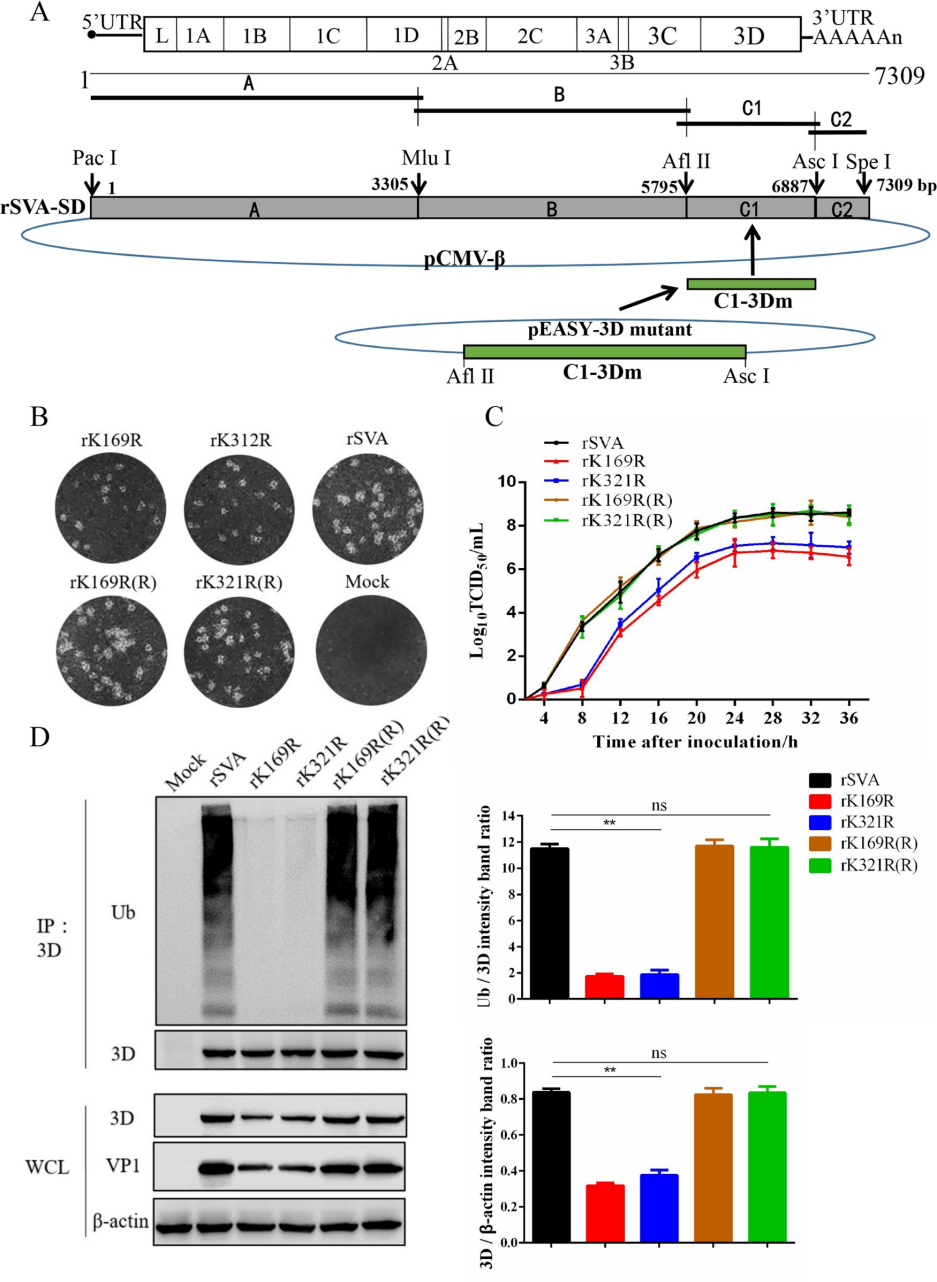
Construction and ubiquitination detection of mutant viruses. (**A**) Construction scheme of full-length cDNA clones containing mutations in 3D and two rescued SVA strains. (**B**) BHK-21 cells infected with 100 TCID_50_ parental rSVA or mutants were used for one-step growth curves. (**C**) Plaque morphology in BHK-21 cells of rescued viruses. (**D**) Co-IP and Western blotting of infected BHK-21 cells. The densitometric analysis of Ub/3D or 3D/β-actin protein levels are depicted as relative intensities. Values are presented as the mean ± SD from three independent experiments. *, P < 0.05; **, P < 0.01.

To evaluate the importance of K169 and K321 on SVA replication, recombinant viruses containing the mutations K169R, K321R, and K169R/K321R and their revertants were constructed (Fig 10A). As seen in Fig 10B, plaque morphologies of the viral mutants (rK169R, rK321R) and their revertants (rK169R(R), rK321R(R)) were similar to rSVA, although the mutant viruses grew more slowly and their titers were significantly lower (Fig 10C). In addition, the levels of 3D ubiquitination in the mutants were nearly undetectable, whereas 3D ubiquitination in rSVA and the revertants were similar and robust (Fig 10D). These results indicate that ubiquitination of the 3D protein at K169 and K321 is important for SVA replication.

## Discussion

In this study we found that UBE2L6, the E2 ubiquitin/ISG15-conjugating enzyme, significantly promoted the replication of SVA in BHK-21 cells by polyubiquitinating the viral 3D protein. This UBE2L6 mediated polyubiquitination depends on its cysteine at position 86 and the lysines at position 169 and 321 on the 3D protein. Our data demonstrates that UBE2L6 plays a substantive role in SVA infection of host cells through posttranslational modification pathways. UBE2L6, as a ubiquitin binding enzyme, probably functions in multiple ubiquitylating pathways with different E3s. Changes in its expression could influence cell vitality and the ability to support SVA replication. Here, our results showed that there was no significant difference in cell activity between UBE2L6 knockout cell lines and wild-type cells. These results are similar to those reported by Leto *et al*. in cells deleted of the E2s UBE2D3 and UBE3C [27].

The ubiquitin-proteasome system (UPS) impacts myriad viral infection processes. For example, E3 ubiquitin ligase pRNF114 interacts with the NS4B protein of swine fever virus and mediates K27-linked polyubiquitination of NS4B, leading to its degradation [28]. E3 ligase ASB8 degrades the innate immune protein IKKβ kinase and stabilizes Nsp1α of PRRSV during infection [29]. Here, we found that during SVA infection UBE2L6 was significantly up-regulated leading to significantly increased levels of 3D ubiquitination. Ubiquitination enzymes are of three general classes: ubiquitin-activating, ubiquitin-conjugating, and ubiquitin-protein ligating. UbcH9, a conserved Ub-conjugating enzyme, also associated with cell development, proliferation, and the cellular antioxidant defense system [30], was shown to be SUMO-specific E2 for signaling by the SUMO protein [31,32]. Reports have suggested that UBE2L6 acts as an ISG15-conjugating enzyme, playing a regulatory role in cellular processes through ISGylation [33–35]. UBE2L6 inhibits human cytomegalovirus (HCMV) growth by downregulating viral gene expression and virion release in HF cells. HCMV develops several strategies to disarm ISGylation-mediated antiviral activity. IE1 reduces ISG15 transcription and UL26 inhibits protein ISGylation [36]. In this study, we found that UBE2L6 only interacts with the SVA-3D protein. It is worth to exploring the SVA and cellular proteins that are targets of ISGylation and whether UBE2L6 regulates SVA replication by ISGylation.

The 3D protein of SVA is an RNA-dependent RNA polymerase, as in other picornaviruses it is essential for viral replication [37]. The microRNAs targeting the 3D polymerase of FMDV effectively inhibit virus replication *in vitro* [38]. Monoclonal antibodies targeting the 3D protein of enterovirus 71 (EV71) affect its RdRp activity and inhibit virus replication [39]. Hao *et al*. reported that EV71 3D protein of interacts with METTL3, causing N6 methyladenosine to modify 3D and promote virus replication. Interestingly, ubiquitination is also involved in this process, along with SUMOylation, to stabilize 3D protein [40]. In this study, we found that UBE2L6 is utilized by SVA to enhance replication via UBE2L6-mediated ubiquitination of the viral RdRp, 3D, which serves to stabilize 3D. The 3C protein of SVA has been reported to negatively regulate the innate immune response through deubiquitination, thereby promoting virus replication [41]. These results demonstrate that SVA has various strategies to utilize the host UPS to help it better proliferate in infected cells. Ubiquitination also plays an important role in regulating Coxsackie virus CVB3 replication. As reported by Si, X *et al*., RdRp 3D is ubiquitinated during CVB3 infection, although which ubiquitination enzymes are involved is unclear [18]. In this study, we demonstrated that UBE2L6 participated in the process of SVA infection and ubiquitinated the 3D protein. Although there are differences between the sequences of SVA-3D and CVB3-3D, the two ubiquitination modification sites (K169 and K321) of the SVA 3D protein discussed in this study are relatively conserved in picornavirus. Therefore, we speculate that UBE2L6 may also participate in the ubiquitination modification of Coxsackievirus CVB3 3D. For another member of the picornavirus family, enterovirus EV71, curcumin, a traditional antiviral drug, can significantly suppress the synthesis of viral RNA, the expression of viral protein, and the overall production of viral progeny. As reported by Qin, Y *et al*., curcumin decreased the activity of the proteasome which had been increased by viral infection, this in turn led to an increase in the accumulation of cell cycle regulatory proteins p21 and p53 [42]. It was thought that the ubiquitin-proteasome system may be a common feature of picornavirus virus proliferation.

Ubiquitin molecules contain seven lysine residues (K6, K11, K27, K29, K33, K48, and K63) that can form ubiquitin chains on target protein through their lysine residues [43–45]. K48-linked ubiquitin chains mainly cause proteasome degradation of target proteins, while K63-linked ubiquitin chains catalyze repair of DNA damage, stabilize proteins, and activate protein kinases [46–48]. Here, we show that in cells transfected with Ub-K48R and Ub-K63R mutants, UBE2L6-mediated 3D polyubiquitination levels were significantly lower than in cells transfected with wild type Ub. In cells transfected with UBE2L6, levels of endogenous Ub, K48, and K63 ubiquitination of 3D were increased. MG132 treatment induced significantly higher levels of Ub and K48 ubiquitination, but not of K63, which indicates that UBE2L6 modifies the SVA 3D protein by K48 and K63 ubiquitination. Additionally, UBE2L6 controls the stability of 3D by preventing the proteasomal degradation of 3D. These results support the reports that UBE2L3 can catalyze the formation of linear, K11, K48, and K63-based chains on other substrates, and ultimately maintains the stability of those substrates [49–53]. Previous reports also have identified UBE2L3 as catalyzing the conjugation of p27Kip1 to heterogeneous ubiquitin chains and protecting them from degradation [54]. RNF4 forms K11/K33 ubiquitin chain conjugates of β-catenin that inhibit degradation [55], and SCFFbl12 forms K48/K63 ubiquitin chains inhibiting degradation of p21^Cip1/Waf1^ [56]. Here we found that UBE2L6 inhibits the degradation of SVA 3D by catalyzing the K48/K63 mixed chains. Of course, whether other lysine residues of Ub molecules are also involved in the formation of 3D ubiquitin chains catalyzed by UBE2L6 needs further investigation. By the way, UBE2L3 (UbcH7) is similar to UBE2L6 (UbcH8). The impact of UBE2L3 on SVA replication should be investigated in the future.

Ubiquitin is conjugated to a lysine in the target protein through a three-step enzymatic reaction. It is helpful to characterize the binding site of the target protein to clarify the relationship between UPS and the target protein [57–59]. SVA 3D has 27 lysine residues. Motif analysis predicts that eleven of these (K47, K87, K95, K161, K169, K321, K360, K366, K401, K418 and K431) are potential ubiquitination sites. The SUMOylation sites of EV71 3D protein (K159 and L150/D151/L152) are located in the center or adjacent regions of the protein [60]. We speculated that the ubiquitination sites on SVA 3D were similarly located. Our results showed that the lysines at 169 and 321 are essential for 3D ubiquitination. Two recombinant viruses with mutations at residues 169 or 321 were constructed and rescued. Here, we found that either the rK169R or rK321R mutation abolished 3D ubiquitination in infected cells. A single C36S or C60S mutation of TRIM35 is also sufficient to abolish the E3 ligase activity of TRIM35, blocking the polyubiquitination of TRAF3 [61]. Therefore, we speculate that there is cross talk between the K169 and K321 residues, such that K169 and K321 are both required for 3D polyubiquitination. Future studies will be necessary to elucidate all ubiquitination modification sites of 3D by immunoprecipitation and mass spectrometry analysis.

Functioning as a polymerase, the RdRp domain of 3D is responsible for RNA synthesis in some positive-strand RNA viruses [62,63]. Modifications of RdRp may affect polymerase activity and virus replication. For example, the K159 residue in polymerase 3D of EV71 is located within the RdRp catalytic motif. Its SUMO modification stabilizes 3D and facilitates viral replication. The K159A mutation decreased polymerase activity *in vitro*, and the recombinant virus could not be rescued. In contrast, the K159R mutation retained a WT level of polymerase activity, and the corresponding recombinant virus replicated as well as its parent [60]. In this study, we found that the K169 ubiquitination site is located within the RdRp catalytic motif of SVA 3D and is highly conserved in Picornaviridae viruses. The 3D ubiquitination-deficient SVA strain rK169R is replication deficient compared to its parent. The K321 ubiquitination site, which is relatively more conserved in picornaviral RNA polymerases compared to K169, is not located in the polymerase activity region. The rescued recombinant rK321R also replicated at lower levels than its parent. The defect in replication of the SVA mutant is likely due to reduced polymerase activity and/or the impairment of ubiquitination of 3D. This hypothesis merits future investigation.

**Fig 11:**
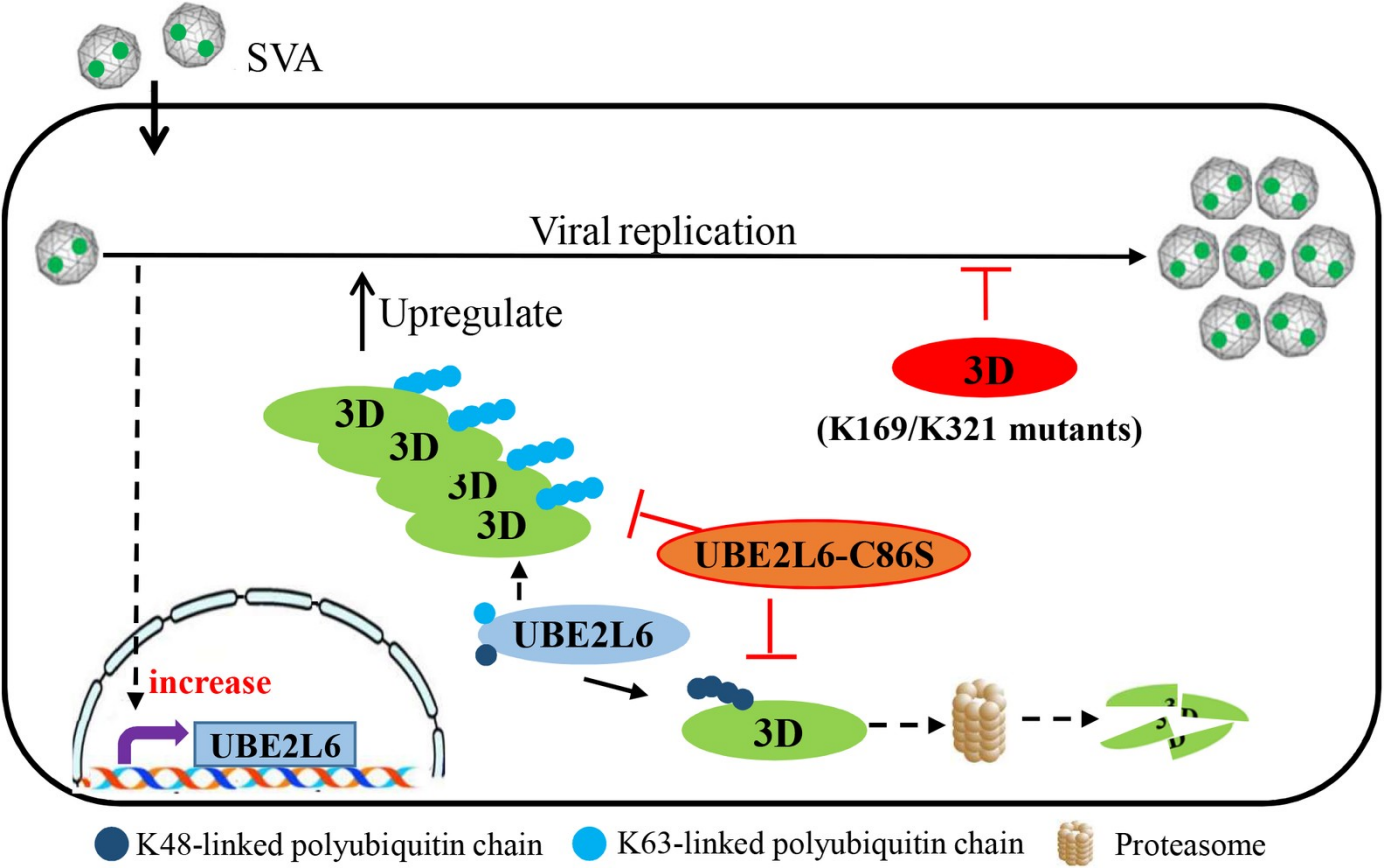
Model of UBE2L6-mediated promotion of SVA replication. SVA infection upregulates the expression of UBE2L6, an E2 ubiquitin-binding enzyme, and UBE2L6 interacts with viral protein 3D and mediates K48/K63 chains to improve the stability of SVA 3D. In UBE2L6, the mutation C86S results in the loss of UBE2L6-mediated ubiquitination of 3D, and in 3D, the mutations K169R and K321R also result in unubiquitinated 3D, as these are the sites of UBE2L6-mediated ubiquitination. Any of these mutations result in inhibition of SVA replication.

In summary, we found that UBE2L6 is upregulated in SVA infected cells and ubiquitinates viral RdRp 3D and inhibits its degradation, thereby promoting SVA replication. The cysteine residue at position 86 of UBE2L6 is necessary for UBE2L6-mediated 3D polyubiquitination, and the lysine residues at positions 169 and 321 in 3D are the sites of UBE2L6-mediated ubiquitination (Fig 11). These findings provide insight into the role of the host UPS during SVA replication and suggest potential targets for controlling SVA infection.

## Materials and methods

### Cells, viruses, and reagents

Swine testicular (ST) cells and baby hamster kidney-21 (BHK-21) cells were cultured in Dulbecco’s modified Eagle’s medium (DMEM) (Invitrogen, USA) at 37°C in a humidified 5% CO_2_ atmosphere. SVA strain isolate SVV-CH-SD (GenBank accession no. MH779611) was passaged in BHK-21 cells and stored in our laboratory. pCAGGS vector that had been modified to contain 3' FLAG-, HA-, or Myc-tag was kindly provided by Dr. Zhigao Bu at the Harbin Veterinary Research Institute, Chinese Academy of Agricultural Sciences [64]. Plasmid HA-Ub was kindly provided by Dr. Yingjuan Qian at the MOE Joint International Research Laboratory of Animal Health and Food Safety, College of Veterinary Medicine, Nanjing Agricultural University, Nanjing, China. Plasmids HA-Ub-K48 (of the seven Lys residues, only K48 remained) and HA-Ub-K63 (of the seven Lys residues, only K48 remained) were kindly provided by Dr. Haixue Zheng at the Lanzhou Veterinary Research Institute, Chinese Academy of Agricultural Sciences [41]. HA-Ub was used as the backbone for construction of the HA-Ub-K48R and HA-Ub-K63R mutants. In each of these mutants only one of the seven Lys residues are mutated. Anti-HA, anti-Flag, and anti-Myc antibodies were purchased from Abmart, China, Anti-Ub, Anti-K48-Ub, and Anti-K63-Ub from Cell Signaling Technology, USA, and anti-β-actin from proteintech Biotechnology, USA. The monoclonal antibodies (MAb) for VP1 and 3D were made in our laboratory. HRP-labeled goat anti-mouse, mouse anti-goat antibodies and MG132 were purchased from Beyotime, China.

### Protein preparation, iTRAQ labeling, and LS-MS/MS

Mock or SVA infected ST cells were cultured in 6-well plates for 12 and 24 hours. The cells were then washed with cold PBS and lysed by sonication in STD lysate buffer (4% SDS, 100 mM DTT, 150 mM Tris-HCl pH 8.0). The detergent, DTT, and other low-molecular-weight components were removed using UA buffer (8 M Urea, 150 mM Tris-HCl pH 8.0) by repeated passage through 30 kD Microcon ultrafiltration units. The filters were incubated with 100 μl UA buffer containing 0.05 M iodoacetamide for 20 min in darkness, thee washed three times with 100 μl UA buffer and twice with 100 μl DS buffer (50 mM triethylammoniumbicarbonate at pH 8.5). The protein suspensions were digested overnight at 37°C with 40 μl DS buffer containing 2 μg trypsin, and the resulting peptides were collected as a filtrate. Peptides were labeled using an iTRAQ Reagent 8-plex kit according to the manufacturer’s instructions. Each fraction was reconstituted in solution A and then introduced by an autosampler into a C18 capture column in a LC-20AD nano-HPLC instrument. Samples were eluted with a gradient of solvent B. Mass spectrometry was performed using the TripleTOF 5600 System platform [65].

### Plasmid construction

Genes encoding the SVA proteins L, VP1, VP2, VP3, VP4, 2B, 2C, 3A, 3C, and 3D were amplified from SVA strain SVV-CH-SD. The PCR fragments were digested with restriction enzymes and cloned into a pCAGGS vector that had been modified to contain a 3’ FLAG-tag. Similarly, genes encoding UBE2L6 were amplified from ST cell genomic cDNA and cloned into pCAGGS that had been modified to contain a 3’ FLAG-, HA-, or Myc-tag. UBE2L6 protein mutants, generated by PCR site-directed mutagenesis of pCAGGS-UBE2L6-Myc, were designated pCAGGS-UBE2L6(C86S), (C98S), and (C102S). The 3D protein mutants, generated by PCR site-directed mutagenesis of pCAGGS-3D, were cloned into the pCAGGS vector using EcoR I and Kpn I. These clones were designated pCAGGS-3D(K161R), (K169R), (K321R), (K366R), and (K418R). All constructs were validated by DNA sequencing. Primers are shown in S1 Table.

### Construction of a UBE2L6 knockout cell line

Three sgRNA sequences were designed using the online CRISPR design tool (https://www.benchling.com) to target the second and third exons of *UBE2L6* (Fig 4A). A Bbs I restriction site was added at the sgRNA 5' terminus during synthesis. Double-stranded nucleotide fragments were obtained by annealing the synthesized sgRNA oligonucleotides at 95°C for 5 minutes, and then the temperature was ramped from 95°C to 25°C at a rate of 0.1°C/s. The annealed fragments were digested with Bbs I and cloned into the CRISPR-Cas9 vector pX-459 to generate pX-459-UBE2L6-sgRNA1, pX-459-UBE2L6-sgRNA2, and pX-459-UBE2L6-sgRNA3.

BHK-21 cells at 50% confluence in 12-well plates were transfected with empty vector control or recombinant plasmid using Lipofectamine3000 transfection reagent according to the manufacturer’s instructions. 48 hours after transfection, the transfection media was replaced with fresh cell culture medium (FBS: 10%, puromycin: 8 mg/mL). In order to isolate single-cell clones, cells were grown to 50% confluency under puromycin selection, diluted to concentrations of 100 and 10 cells/mL, and aliquoted into the wells of 96-well cell culture plates. After incubation for about 1 week, colonies formed from single cells could be identified and were harvested for genomic analysis. Genomic DNA extracted from these clones was used as template to amplify target region sequences and evaluate knockout results. Detection primers for the first and second exons (S2 Table) were used to amplify target region sequences. The resulting amplicons were cloned into the vector pEASY-Blunt-Simple (TransGen Biotech, China) for sequencing. Cells containing deletions, additions, or mutations in *UBE2L6* were cultured and expanded. Western blotting with UBE2L6 endogenous antibodies was used to identify the UBE2L6 protein knockout [66].

### Construction of infectious SVA cDNA clones

To construct full-length cDNA clones, four pairs of primers were designed using the SVA-CH-SD strain (GenBank accession no. MH77961) sequence as template. Four separate fragments (A, B, C1, and C2), each with a unique restriction enzyme site obtained by synonymous mutation [67], were amplified using Pfu DNA Polymerase (Novoprotein, China). The resulting full-length cDNA clone, designated pCMV-rSVA, contained a *Mlu* I site at the junction of fragments A and B, an *Afl* II site at the junction of fragments B and C1, and an *Asc* I site at the junction of fragments C1 and C2. Because the restriction sites *Mlu* I, *Afl* II, and *Asc* I were obtained by synonymous mutation, we chose *Afl* II as a molecular marker for differentiating cloned virus from parental SVV-CH-SD (Fig 10A).

To generate the mutations, the *Afl* II/*Asc* I fragment containing the C1 fragment of SVA was amplified from plasmid pCMV-rSVA-C by PCR. The PCR product was cloned into pEASY-Blunt-Simple (TransGen Biotech, China), and designated as pEASY-C1. Site-directed mutations in 3D were constructed using pEASY-C1, and designated as pEASY-C1-3Dm. The *Afl* II/*Asc* I fragment of the pCMV-rSVA backbone was replaced by the corresponding region from pEASY-C1-3Dm. The full-length clone containing the mutation was designated as pCMV-rSVA/3Dm (Fig 6A). To construct revertants, C1-3Dm(R) was obtained by site-directed mutagenesis using pCMV-rNJ08/2Cm as the template. The *Afl* II/*Asc* I fragment of pCMV-rSVA was replaced with C1-3Dm(R), generating pCMV-rSVA/3Dm(R). All constructs were confirmed by DNA sequencing. Plasmids were purified using a FastPure plasmid mini kit (Vazyme Biotech, China). All primers used for generating amino acid substitution mutants and recombinant viruses are listed in S3 Table.

To rescue viruses, plasmids containing full-length viral cDNAs were transfected into BHK-21 cells using Lipofectamine 3000 according to the manufacturer’s protocol. When about 90% of cells exhibited CPE, the overlay media was collected, then serially passaged five times on BHK-21 cells. Stocks from each passage were stored at -80°C. The rescued viruses were designated rSVA, rK169R, rK321R, rK169R(R), and rK321R(R).

### Co-immunoprecipitation and Western blotting

BHK-21 cells were lysed in RIPA buffer containing PMSF. For co-immunoprecipitation (Co-IP), 1 ml of whole cell lysate was incubated with 1 μg of homograft mouse antibody and 20 μl of protein A/G agarose beads (Beyotime, China) for 2 h at 4°C. The lysates were centrifuged at 2500 rpm for 5 minutes. The supernatants were collected and incubated with 2 μg of the appropriate mouse antibody and 20 μl of protein A/G agarose beads overnight on rollers at 4°C. The agarose beads were collected by centrifugation, washed five times with 1 ml of lysis buffer, then suspended in 100 μl lysis buffer. Whole cell lysates and immunoprecipitates were subjected to 10% SDS-PAGE then transferred onto a nitrocellulose membrane. Membranes were blocked in Tris-buffered saline containing 10% nonfat dry milk for 2 h at 37°C, then washed with phosphate-buffered saline containing 0.1% Tween-20 (PBST). Membranes were incubated with primary antibodies for 2 h at 37°C, then washed with PBST and incubated with secondary antibody for 45 min at 37°C. The membranes were washed again and treated with ECL (Thermo Fisher Scientific). Bound proteins were visualized with a Tanon 5200 Chemi-Image system (Biotanon, China).

### Confocal microscopy

BHK-21 cells were transfected with pCAGGS or pCA-UBE2L6-HA for 24 h, then infected with SVA at 0.01MOI. At 16 hours post infection (hpi) cells were fixed with 4% paraformaldehyde for 15 min at RT, permeabilized with 0.1% Triton X-100 for 10 min at 4°C, and then incubated with rabbit anti-HA (1:200) or mouse anti-SVA 3D (1:200) for 2 h at 37°C. Cells were washed with PBS then incubated with Alexa Fluor 488 labeled anti-rabbit (1:250) and Alexa Fluor 494 labeled anti-mouse (1:250) for 1 h at 37°C. Cells were washed again with PBS, stained with 4’,6-diamidino-2-phenylindole (DAPI) for 10 min, and processed for confocal microscopy.

### RNA isolation and qRT-PCR

*IFIT1*, *MX2*, *UBE2L6*, *ISG15*, *RSAD2*, *ANXA6*, *STRBP*, and *SWAP70* mRNA expression was quantitated by qRT-PCR. Following the manufacturer’s protocol, total RNA was extracted from ST cells using a Total RNA Kit I (Omega Bio-Tek). qRT-PCR was performed in an ABI QuantStudio 6 System (Applied Biosystems) using a SYBR-Green RT-PCR Master Mix (Applied Biosystems). β-Actin was used as the reference gene, and all data are expressed as relative fold change (calculated using the 2-ΔΔCT method). All primers for qRT-PCR are presented in S4 Table.

### CHX chase experiment

BHK-21 cells in 24-well plates were co-transfected with pCA-3D-Flag and pCA-UBE2L6-Flag or pCA-UBE2L6-Myc. 24 hours after transfection, the cells were treated with 100 μg/ml of CHX dissolved in dimethyl sulfoxide (DMSO) or with DMSO alone (mock). Cells were collected at 0, 6, and 12 h after treatment and subjected to Western blotting.

### Cell viability assay

Following the manufacturer’s instructions, cell viability was determined using a Cell Counting Kit-8 (CCK-8) (Beyotime). Results are expressed relative to control cells, which are defined as 100% viable.

### Plaque assays

BHK-21 cells were seeded into 6-well plates, when cells reached 100% confluence, they were inoculated with 200 TCID_50_ of virus and incubated for 1 h at 37°C. Cells were then overlaid with DMEM containing 2% heat-inactivated FBS and 1% low-melting-point agarose (Sigma-Aldrich), then incubated for an additional 24 h. To visualize plaques, the cells were overlaid with 1% crystal violet in methanol for 5 h at 37°C.

### Viral growth curves

BHK-21 cells seeded into 24-well plates were incubated with the SVA mutants at 10^2.5^ TCID_50_ for 1 h at 37°C. The inoculum was removed and fresh media was added to each well, at 4, 8, 12, 16, 20, 24, 28, 32, and 36 hpi, the cell supernatants were collected and stored at -80°C for TCID_50_ assay.

### Statistical analysis

All data were analyzed with GraphPad Prism 5.0 using one-way ANOVA or Student’s *t*-test. In graphs, error bars represent standard deviations. P-values are indicated using asterisks: *p < 0.05, **p < 0.01, and ***p < 0.001.

## Supporting information

S1 Table Primers used to generate individual viral protein-expressing plasmids, 3D mutants, UBE2L6 protein-expressing plasmids, UBE2L6 mutants, HA-Ub-K48R and HA-Ub-K63R mutants. (XLSX)

S2 Table Primer for generation of UBE2L6-KO cells. (XLSX)

S3 Table Primers used for construction of an infectious cDNA clone of strain SVV-CH-SD mutants. (XLSX)

S4 Table Primers used for RT-PCR. (XLSX)
